# *PRDX1* gene-related *epi-cblC* disease is a common type of inborn error of cobalamin metabolism with mono- or bi-allelic *MMACHC* epimutations

**DOI:** 10.1186/s13148-021-01117-2

**Published:** 2021-07-02

**Authors:** Catia Cavicchi, Abderrahim Oussalah, Silvia Falliano, Lorenzo Ferri, Alessia Gozzini, Serena Gasperini, Serena Motta, Miriam Rigoldi, Giancarlo Parenti, Albina Tummolo, Concetta Meli, Francesca Menni, Francesca Furlan, Marta Daniotti, Sabrina Malvagia, Giancarlo la Marca, Céline Chery, Pierre-Emmanuel Morange, David Tregouet, Maria Alice Donati, Renzo Guerrini, Jean-Louis Guéant, Amelia Morrone

**Affiliations:** 1 Molecular and Cell Biology Laboratory of Neurometabolic Diseases, Paediatric Neurology Unit and Laboratories, Meyer Children’s Hospital, Viale Pieraccini 24, 50139 Florence, Italy; 2 INSERM, UMR_S1256 Nutrition-Genetics-Environmental Risk Exposure and Reference Centre of Inborn Metabolism Diseases, University of Lorraine and University Hospital Centre of Nancy (CHRU Nancy), Nancy, France; 3 Rare Metabolic Disease Unit, Department of Paediatrics, Fondazione MBBM, Monza, Italy; 4 Mario Negri Institute for Pharmacological Research IRCCS, Bergamo, Italy; 5 Metabolic Unit, Federico II Hospital, Napoli, Italy; 6 Metabolic Disease Unit, Giovanni XXIII Hospital, Bari, Italy; 7 Metabolic Disease Unit, G. Rodolico Hospital, Catania, Italy; 8 Fondazione IRCCS Ca’ Granda Ospedale Maggiore Policlinico, Paediatric Highly Intensive Care Unit, Milan, Italy; 9 Metabolic and Muscular Unit, Meyer Children’s Hospital, Florence, Italy; 10 Newborn Screening, Biochemistry and Pharmacology Laboratory, Meyer Children’s Hospital, Florence, Italy; 11 Department of Experimental and Clinical Biomedical Sciences, University of Florence, Florence, Italy; 12 Aix-Marseille University, INRAE, INSERM, C2VN, Marseille, France; 13 INSERM, UMR_S937, ICAN Institute, Université Pierre et Marie Curie, Paris, France; 14 Department of NEUROFARBA, University of Florence, Florence, Italy

**Keywords:** Secondary epimutation, Promoter hypermethylation, CpG island, Methylmalonic aciduria and homocystinuria, *cblC* type, *cblC* disease, *Epi-cblC*, Expanded newborn screening (NBS)

## Abstract

**Background:**

The role of epigenetics in inborn errors of metabolism (IEMs) is poorly investigated. Epigenetic changes can contribute to clinical heterogeneity of affected patients but could also be underestimated determining factors in the occurrence of IEMs. An epigenetic cause of IEMs has been recently described for the autosomal recessive methylmalonic aciduria and homocystinuria, *cblC* type (*cblC* disease), and it has been named *epi-cblC*. *Epi-cblC* has been reported in association with compound heterozygosity for a genetic variant and an epimutation at the *MMACHC* locus, which is secondary to a splicing variant (c.515-1G > T or c.515-2A > T) at the adjacent *PRDX1* gene. Both these variants cause aberrant antisense transcription and cis-hypermethylation of the *MMACHC* gene promotor with subsequent silencing. Until now, only nine *epi-cblC* patients have been reported.

**Methods:**

We report clinical/biochemical assessment, *MMACHC*/*PRDX1* gene sequencing and genome-wide DNA methylation profiling in 11 cblC patients who had an inconclusive *MMACHC* gene testing. We also compare clinical phenotype of *epi-cblC* patients with that of canonical *cblC* patients.

**Results:**

All patients turned out to have the *epi-cblC* disease. One patient had a bi-allelic *MMACHC* epimutation due to the homozygous *PRDX1*:c.515-1G > T variant transmitted by both parents. We found that the bi-allelic epimutation produces the complete silencing of *MMACHC* in the patient’s fibroblasts. The remaining ten patients had a mono-allelic *MMACHC* epimutation, due to the heterozygous *PRDX1*:c.515-1G > T, in association with a mono-allelic *MMACHC* genetic variant. *Epi-cblC* disease has accounted for about 13% of *cblC* cases diagnosed by newborn screening in the Tuscany and Umbria regions since November 2001. Comparative analysis showed that clinical phenotype of *epi-cblC* patients is similar to that of canonical *cblC* patients.

**Conclusions:**

We provide evidence that *epi-cblC* is an underestimated cause of inborn errors of cobalamin metabolism and describe the first instance of *epi-cblC* due to a bi-allelic *MMACHC* epimutation. *MMACHC* epimutation/*PRDX1* mutation analyses should be part of routine genetic testing for all patients presenting with a metabolic phenotype that combines methylmalonic aciduria and homocystinuria.

**Supplementary Information:**

The online version contains supplementary material available at 10.1186/s13148-021-01117-2.

## Introduction

The role of epigenetics in inborn errors of metabolism (IEMs) is poorly investigated. IEMs are a large and heterogeneous group of genetic disorders caused by single gene defects that disrupt normal metabolism. Many IEMs can be detected by newborn screening (NBS) with significant reduction of mortality and disease burden, if promptly treated [1, 2]. Besides their genetic causes, it is increasingly recognizing that epigenetic changes contribute to clinical heterogeneity of patients affected by IEMs [3–5]. Moreover, routine genetic test of genes responsible for IEMs may be inconclusive in some patients with a clear-cut clinical phenotype. Epigenetics could have an underestimated causal role in these patients. In fact, an epigenetic cause of IEMs, named *epi-cblC*, has been recently reported for methylmalonic aciduria and homocystinuria, cobalamin C type (*cblC* disease; OMIM #277400) [6]*.*

*CblC* disease is the most common inborn error of intracellular vitamin B12 (also called cobalamin, cbl) metabolism with a worldwide prevalence ranging from 1:37,000 to 1:100,000 [7]. It is caused by a deficiency of the MMACHC protein, which is needed to convert vitamin B12 into adenosylcobalamin (AdoCbl) or methylcobalamin (MeCbl), the two cofactors of methylmalonyl-CoA mutase and methionine synthase, respectively [8]. This combined enzymatic defect leads to increased concentrations of methylmalonic acid (MMA) and homocysteine (Hcy) in plasma and urine with normal or decreased concentrations of methionine (Met) in plasma [8]. Propionylcarnitine (C3) and the C3/acetylcarnitine (C2) ratio, combined with the second-tier markers MMA and total Hcy, are the tandem mass spectrometry biomarkers for the early detection of *cblC* disease in expanded NBS programs [9, 10].

*CblC* disease is clinically heterogeneous for signs and symptoms and age of presentation. Depending on the age of presentation, patients have been classified into early-onset (< 1 year) and late-onset (> 1 year) [11]. More recently, three clinical forms have been distinguished: prenatal, infantile and non-infantile [8].

*CblC* disease is caused by recessive pathogenic variants in the *MMACHC* gene (Gene ID: 25974; OMIM *609831, location: 1p34.1) [11] in which 115 disease-causing variants have been reported so far (http://www.biobase-international.com/product/hgmd), some of them clustering according to ethnicity [12]. The most common is c.271dupA p.(Arg91Lysfs*14) which represents at least 30% of mutant alleles in Europe [12]. Next-generation sequencing (NGS) permits differential diagnosis among the spectrum of cobalamin disorders and can also identify copy number variations (CNVs) that account for about 6% of reported *MMACHC* disease-causing variants [8]. However, some cases remain without a conclusive molecular diagnosis. Some of these cases recently have been diagnosed as affected by *epi-cblC* disease [6]. Previously reported e*pi-cblC* patients are compound heterozygous for a genetic variant and a secondary epimutation at the *MMACHC* locus. The secondary epimutation results from a splicing variant in the adjacent *PRDX1* gene (peroxiredoxin 1; Gene ID: 5052; OMIM *176763), corresponding to either a c.515-1G > T or a c.515-2A > T substitution. Both these *PRDX1* variants are located in an acceptor site and cause an aberrant antisense transcription starting from *PRDX1* and encompassing the *MMACHC* gene promoter. Antisense transcription through the promoter of the *MMACHC* gene induces *cis*-hypermethylation of the promoter with subsequent *MMACHC* transcriptional silencing [6].

Herein, we report the molecular analysis of 11 Italian probands, with a diagnosis of methylmalonic aciduria and homocystinuria between 2008 and 2018, who had previously been tested with *MMACHC* gene sequencing without reaching any conclusive molecular diagnosis. Among them, ten turned out to have the *epi-cblC* form of the disease, with compound heterozygosity for an epigenetic and a genetic *MMACHC* variant. One patient had a bi-allelic *MMACHC* epimutation due to the homozygous *PRDX1* c.515-1G > T variant transmitted by both parents.

## Results

### Clinical and biochemical data

The clinical and metabolic findings of the *epi-cblC* patients are shown in Additional file 1: Tables S2 and S3. All patients had methylmalonic acidemia and homocystinuria and an inconclusive molecular diagnosis for the *MMACHC* gene. In two patients (Pts 3 and 9), disease onset was probably in the prenatal period, as an intrauterine growth restriction was observed. Common symptoms at onset in the young patients included failure to thrive, vomiting, hypotonia, respiratory distress and hematologic abnormalities. Recurrent infections, developmental delay, and ocular disease were frequent later manifestations. Maculopathy was ascertained in seven young patients, in five occurring within the first year of life. Haemolytic uremic syndrome occurred in two patients (Pts 8 and 9). Pt 8 developed anaemia and acute renal failure at 1 month of life which required haemodialysis. Pt 9 developed anaemia with high levels of serum lactate dehydrogenase and proteinuria in the first few days of life.

A diagnosis of combined methylmalonic acidemia and homocystinuria was made by NBS in four patients (Pts 2, 7, 9 and 11) and after a clinical/biochemical assessment in the remaining seven patients. One of the patients recognized clinically (Pt 1) was negative at NBS for metabolic disorders but underwent metabolic investigation because of hyperalaninaemia detected on his DBS at birth and early symptomatology. Pt 3 developed symptoms before his NBS results were available. Four patients (Pts 4–6 and 8) were born before the NBS was introduced and one patient was an adult when diagnosed (Pt 10). Three of the patients identified by NBS (Pts 2, 9 and 11) were promptly confirmed to have methylmalonic aciduria, thanks to a second-tier test performed within the sixth day of life which determined MMA on the DBS.

The adult patient (Pt 10) had a complex medical history, mainly characterized by antiphospholipid syndrome, renal symptoms and thrombotic events. At 53 years of age, he received a diagnosis of homocystinuria. It was only after urinary organic analysis at the age of 63 years that increased MMA was detected and that a final diagnosis of methylmalonic acidemia and homocystinuria was made. This patient never developed the typical retinal alterations of *cblC* disease. His only ocular signs were xeropthalmia associated with astigmatism in the context of systemic lupus erythematosus and dry eyes, and cataract, probably related to his age.

With the exception of Pt 4, who died in the second month of life during an acute metabolic crisis, all patients have been treated with hydroxocobalamin, levocarnitine, betaine and folates since diagnosis. The levels of metabolic markers in 9/10 treated patients show a good response to long-term therapy; the exception is Pt 7 who still has unsatisfactory levels of MMA and Hcy. Therapy has not, however, prevented neurological and ocular symptoms, even in patients identified by NBS and treated early. This is in line with observations in canonical *cblC* patients [12, 13].

### Routine molecular analysis for methylmalonic acidemia and homocystinuria

Sanger sequencing of the *MMACHC* gene identified a heterozygous genetic variant in 10/11 patients. No pathogenetic variant was identified in the *MMACHC* gene of Pt 11. Molecular findings of the *epi-cblC* patients are summarized in Table 1. All variants identified in the *MMACHC* gene were previously reported [11, 14]. The c.271dupA p.(Arg91Lysfs*14) and the c.666C > A p.(Tyr222*) were found in 5 and 2 patients, respectively. Pt 10 was heterozygous for the *MMACHC:*c.617G > A p.(Arg206Gln) variant [14] and homozygous for the thermolabile polymorphism NM_005957.5:c.665C > T p.(Ala222Val) of the *MTHFR* gene [15]. NGS analysis excluded other genetic defects of cbl metabolism and CNVs in Pts 9, 10 and 11.

**Table 1:** Pathogenic variants identified in the *MMACHC*/*PRDX1* genes of *epi-cblC* patients

Pt	Italian origin	Diagnosis (age)	Variant 1	Origin of variant 1	Variant 2	Origin of variant 2	Epimutation in *MMACHC*	Age at *epi-cblC* molecular diagnosis	Onset
1	Central	C/B (10 d)	*MMACHC*:c.271dupA p.(Arg91Lysfs*14)	Paternal	*PRDX1*:c.515-1G > T	Maternal	Het	10 y	Early
2	Central	NBS (6 d)	*MMACHC*:c.666C > A p.(Tyr222*)	Paternal	*PRDX1*:c.515-1G > T	Maternal	Het	8 y	Early
3	Southern	C/B (16 d)	*MMACHC*:c.271dupA p.(Arg91Lysfs*14)	Paternal	*PRDX1*:c.515-1G > T	Maternal	Het	11 y	Early
4^a^	Southern	C/B (2 m)	*MMACHC*:c.666C > A p.(Tyr222*)	Maternal	*PRDX1*:c.515-1G > T	Paternal	Het	ND	Early
5	Northern	C/B (2 m)	*MMACHC*:c.331C > T p.(Arg111*)	Paternal	*PRDX1*:c.515-1G > T	Maternal	Het	10 y	Early
6	Northern	C/B (6 m)	*MMACHC*:c.481C > T p.(Arg161*)	Paternal	*PRDX1*:c.515-1G > T	Maternal	Het	6 y	Early
7	Southern	NBS (1 m)	*MMACHC*:c.271dupA p.(Arg91Lysfs*14)	Paternal	*PRDX1*:c.515-1G > T	Maternal	Het	7 y	Early
8	Southern	C/B (1 m)	*MMACHC*:c.271dupA p.(Arg91Lysfs*14)	Maternal	*PRDX1*:c.515-1G > T	Paternal	Het	18 y	Early
9	Northern	NBS (4 d)	*MMACHC*:c.271dupA p.(Arg91Lysfs*14)	Maternal	*PRDX1*:c.515-1G > T	Paternal	Het^b^	2 y	Early
10	Northern	C/B (63 y)	*MMACHC*:c.617G > A p.(Arg206Gln)	ND	*PRDX1*:c.515-1G > T	ND	Het	75 y	Late
11	Central	NBS (4 d)	*PRDX1*:c.515-1G > T	Paternal	*PRDX1*:c.515-1G > T	Maternal	Hom	7 y	Early

C/B, diagnosis of methylmalonic aciduria and homocystinuria performed after a clinical/biochemical assessment; d, days; m, months; het, heterozygous; hom, homozygous; NBS, diagnosis of methylmalonic aciduria and homocystinuria performed by expanded newborn screening; ND, not determined; y, years

^a^We identified the same genotype of Pt 4 in an abortion specimen of this family. Such foetus was biochemically affected

^b^Methylation analysis of this patient has been previously reported by Gueant et al. [6]

### Biochemical phenotyping, *PRDX1* sequencing and *MMACHC* expression analysis for a conclusive diagnosis of *epi-cblC*

**Fig. 1:**
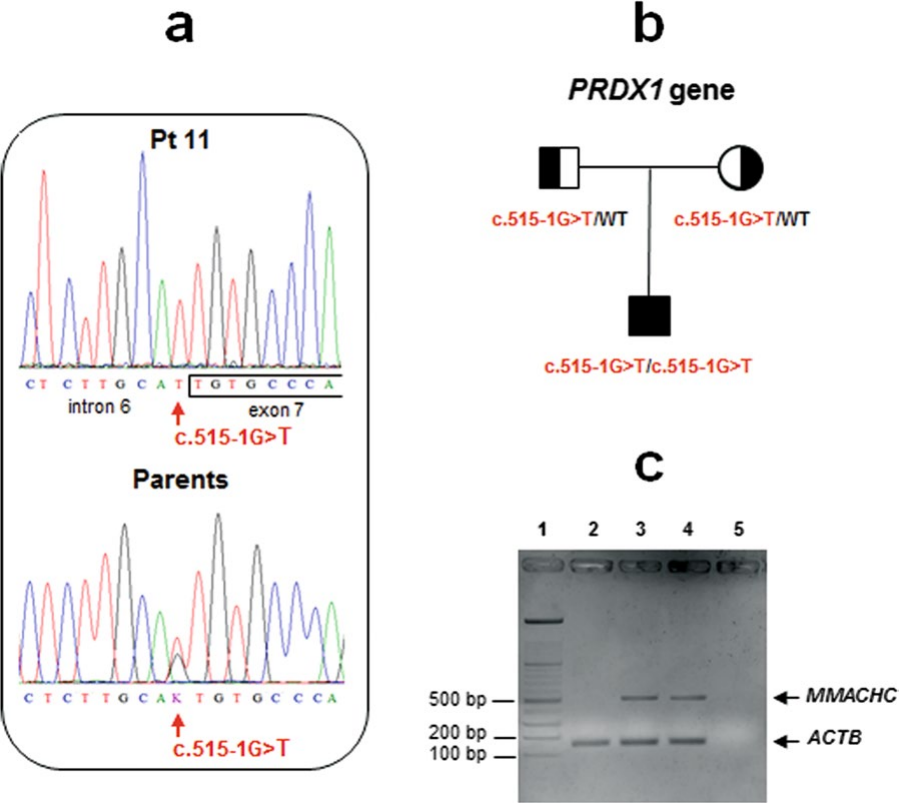
Molecular results of the homozygous *PRDX1* patient (Pt 11). **a** *PRDX1* Sanger sequencing showing the c.515-1G > T pathogenic variant in DNA samples of Pt 11 (at a homozygous level) and his parents (at a heterozygous level). Vertical arrows indicate the position of the mutated nucleotide in the patient and the corresponding nucleotide in his parents. **b** Pedigree of the family. **c** Multiplex RT-PCR showing amplification of *MMACHC* and *ACTB* cDNAs (fragments: 512 bp and 174 bp, respectively). Lane1: molecular marker, lane 2: Pt 11, lane 3, 4: normal controls, and lane 5: no cDNA template

The biochemical phenotyping of fibroblasts from Pts 1, 2, 10 and 11 suggested an intracellular defect of cobalamin metabolism. Complementation analysis clearly indicated that these four patients belonged to the *cblC* complementation group. Sequencing analysis of the *PRDX1* gene identified the c.515-1G > T variant at a heterozygous state in all the patients (10/11) who also harboured an *MMACHC* genetic variant at a heterozygous level (Table 1). With the exception of the 63-year-old patient (Pt 10), *MMACHC/PRDX1* gene sequencing extended to parents of these probands confirmed the allelic segregation of the mutant alleles in all of them. In Pt 11, we found the *PRDX1* c.515-1G > T variant at a homozygous state with no genetic variants in *MMACHC* and any other genes of cobalamin metabolism (Fig. 1a). Both parents of Pt 11 were heterozygous for this *PRDX1* gene variant (Fig. 1a). Family pedigree of Pt 11 is shown in Fig. 1b. Multiplex RT-PCR assays of mRNA from fibroblasts of Pt 11 did not detect any *MMACHC* transcript, compared to results obtained in two healthy controls (Fig. 1c). This result suggested that a bi-allelic epimutation could produce the complete silencing of *MMACHC*.

### Epigenome-wide association study

**Fig. 2:**
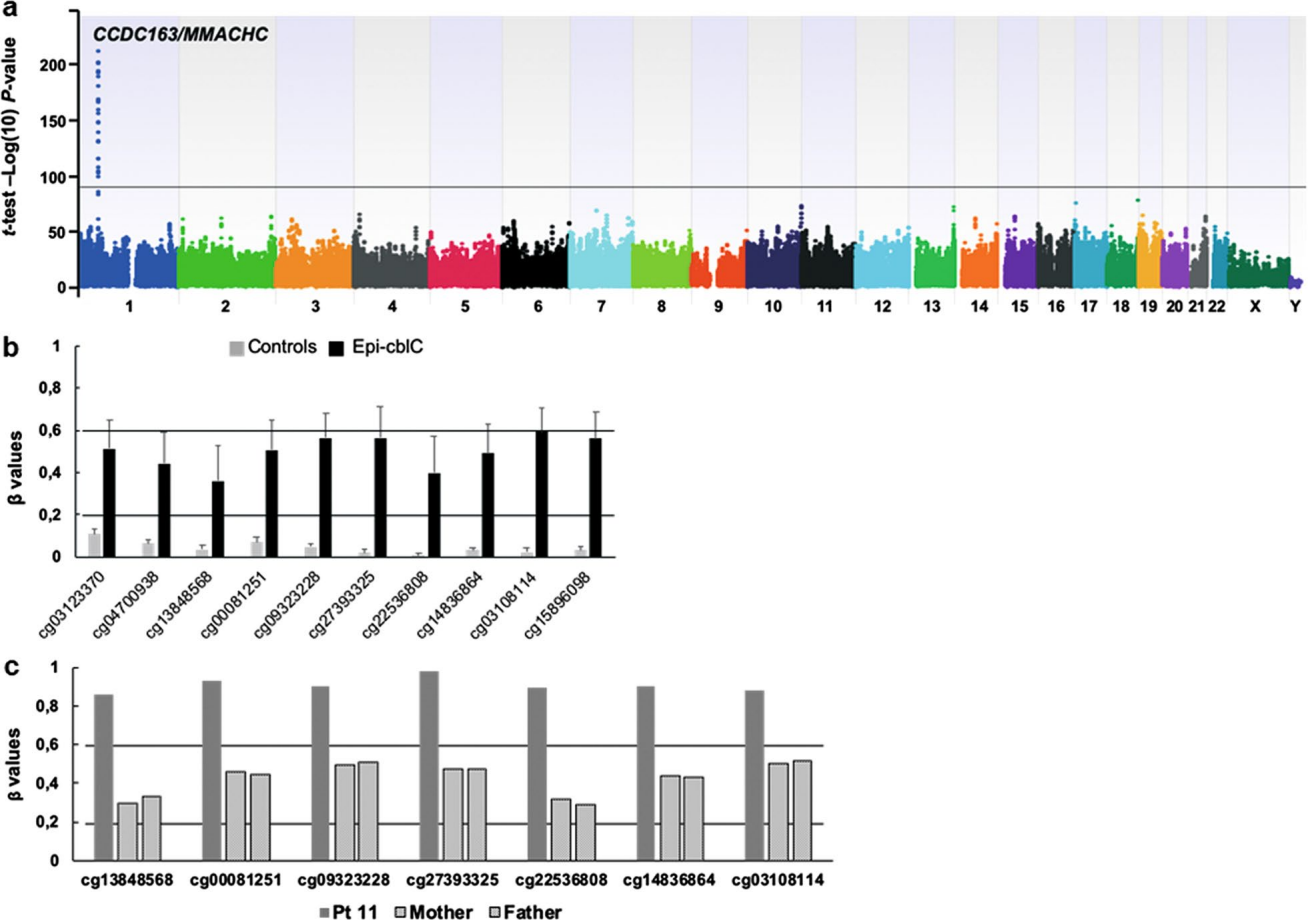
Analyses of DNA methylation in the 11 *epi-cblC* patients. **a** Epi-Manhattan plot reporting the epigenome-wide association study comparing the 11 *epi-cblC* subjects with controls. The −log10 *P*-value reports on the *t*-test comparing the β values of *epi-cblC* subjects and controls. The horizontal line indicates a *P*-value threshold of 1 × 10^–90^. The top significant locus corresponds to the CpG island (CpG:33) on the *CCDC163*/*MMACHC* bidirectional promoter in chromosome 1. **b** Methylation levels of the *CCDC163*/*MMACHC* bidirectional promoter in the *epi-cblC* patients carrying the heterozygous *PRDX1*:c.515-1G > T variant and controls. The horizontal lines correspond to β value thresholds of 0.2, below which the CpG probe is considered to be fully unmethylated. Above 0.6 the CpG probe is considered as fully methylated. A β value between 0.2 and 0.6 indicates a hemimethylated CpG probe. **c** Methylation levels of the *CCDC163*/*MMACHC* bidirectional promoter in the homozygous *epi-cblC* patient (Pt 11) and his parents. All the reported CpG probes exhibit a hemimethylated profile in the parents and a full-methylated profile in the index case harbouring the *PRDX1* splice variant (c.515-1G > T) at the homozygous state

All the DNA methylome profiles of the analysed subjects were of high quality and were used in statistical analyses (Additional file 1: Fig. S1). As shown in the epi-Manhattan plot (Fig. 2a), the epigenome-wide association study retrieved a top significant locus in chromosome 1 at the CpG island CpG:33 on the *CCDC163/MMACHC* bidirectional promoter. All the CpG probes located in CpG island CpG:33 were fully unmethylated among controls and hemimethylated among the *epi-cblC* patients carrying the heterozygous *PRDX1*:c.515-1G > T variant (Table 2 and Fig. 2b). Instead, the *epi-cblC* proband with the bi-allelic epimutation of the CpG island CpG:33 caused by the homozygous *PRDX1* c.515-1G > T splice variant (Pt 11) exhibited a full-methylated profile of all the CpG probes, while his parents exhibited a hemimethylated profile (Fig. 2c).

**Table 2:** Methylation profiles of the *CCDC163/MMACHC* locus in *epi-cblC* subjects harbouring the heterozygous *PRDX1*:c.515-1G > T variant and controls

CpG probe	Chr	Position^a^	CpG island CpG:33	Mean β-value, *epi-cblC*	Mean β-value, controls	*t*-test, *P*-value	*t*-test, Bonf. *P*-value	*t*-test, FDR *P*-value	Smoothed *t*-test, *P*-value
cg03123370	1	45965343	5' end of the CpG:33	0.51	0.11	1.43 × 10^–133^	6.46 × 10^–128^	1.69 × 10^–130^	1.66 × 10^–100^
cg04700938	1	45965449	5' end of the CpG:33	0.44	0.06	4.98 × 10^–130^	2.25 × 10^–124^	5.16 × 10^–127^	6.92 × 10^–109^
cg13848568	1	45965625	Inside CpG:33	0.36	0.04	3.54 × 10^–105^	1.60 × 10^–99^	1.61 × 10^–102^	2.02 × 10^–131^
cg00081251	1	45965679	Inside CpG:33	0.51	0.07	5.99 × 10^–148^	2.71 × 10^–142^	1.09 × 10^–144^	1.12 × 10^–149^
cg09323228	1	45965727	Inside CpG:33	0.57	0.05	2.02 × 10^–203^	9.15 × 10^–198^	1.58 × 10^–199^	7.37 × 10^–157^
cg27393325	1	45965846	Inside CpG:33	0.56	0.02	7.18 × 10^–194^	3.25 × 10^–188^	4.57 × 10^–190^	2.53 × 10^–169^
cg22536808	1	45965870	Inside CpG:33	0.40	0.00	1.03 × 10^–133^	4.68 × 10^–128^	1.24 × 10^–130^	8.75 × 10^–182^
cg14836864	1	45965990	Inside CpG:33	0.49	0.03	7.49 × 10^–184^	3.39 × 10^–178^	3.49 × 10^–180^	8.89 × 10^–161^
cg03108114	1	45966048	Inside CpG:33	0.59	0.02	2.77 × 10^–217^	1.25 × 10^–211^	2.73 × 10^–213^	7.94 × 10^–132^
cg15896098	1	45966115	3' end of the CpG:33	0.56	0.03	2.11 × 10^–192^	9.54 × 10^–187^	1.29 × 10^–188^	2.67 × 10^–105^

Chr, chromosome; Bonf, Bonferroni; FDR, false discovery rate

^a^Position according to CRCh37

### Estimation of *epi-cblC* prevalence and *PRDX1* c.515-1G > T allele frequency

From November 2001 to date, 600,387 newborns have been analysed by the Tuscany-Umbria NBS program at Meyer Children’s Hospital. Twenty-three newborns were diagnosed with *cblC* disease, including three (13%) who had an *epi-cblC* disease (Pts 1, 2 and 11). Hence, the birth prevalence of *cblC* and *epi-cblC* diseases in Tuscany and Umbria could be estimated at around 1:26,000 and 1:200,000, respectively (Additional file 1: Fig. S2a). In the cohort of total cases with *cblC* phenotype and conclusive molecular diagnosis (104 probands), *epi-cblC* disease accounted for 11% of cases (11/104 probands) (Additional file 1: Fig. S2b). In this cohort, 23 different genetic variants in the *MMACHC* gene have been identified. The allele frequency of the common variants is shown in Additional file 1: Fig. S2c. As expected, *MMACHC*:c.271dupA was the most common variant, accounting for about 54% of mutated alleles causing *cblC*. *PRDX1*:c.515-1G > T is the second most frequent disease-causing variant in our cohort with an estimated allele frequency of about 6% (12/208 alleles) (Additional file 1: Fig. S2c).

### Comparison of clinical phenotype between *epi-cblC* and canonical *cblC* patients

Clinical comparison of our *epi-cblC* patients and canonical *cblC* patients, belonging to a larger cohort reported by Huemer et al., showed that the clinical manifestations of *epi-cblC* patients were similar to those of canonical *cblC* patients (Table 3). However, by restricting the comparison to our *MMACHC*:c.271dupA homozygotes from NBS, we found that the clinical phenotype of the *epi-cblC* homozygote appeared to be more severe than the c.271dupA homozygotes (Additional file 1: Table S4). Metabolic findings in the *epi-cblC* homozygote resembled those of the *MMACHC*:c.271dupA homozygotes, although a lower value of plasma methionine was detected in the *epi-cblC* homozygote (2.6 μmol/l compared to the mean value of 7.1 in the c.271dupA homozygotes) (Additional file 1: Table S4).

**Table 3:** Comparison of signs and symptoms in *epi-cblC* and *canonical-cblC* patients

	*Epi-cblC*	*Canonical-cblC*^a^
*Number of reported cases*	11 (this study)	169
*Eating disorders/failure to gain weight*		
Small for gestational age	++ [2 Pts]	+
Feeding difficulties, failure to thrive	++++ [9 Pts]	+++
*Nervous system*		
Decreased consciousness and/or apnoea	++++ [7 Pts]	++
Seizures	+++ [5 Pts]	+++
Ataxia	−	+
Movement disorder and/or abnormal muscle tone	++++ [9 Pts]	+++
Peripheral neuropathy/subacute degeneration of spinal cord	(+)	++
Hydrocephalus	−	++
Visual impairment (retinopathy, optic atrophy)	++++ [9 Pts]	+++
Developmental disorder/cognitive impairment	++++ [8 Pts]	+++
Behavioural/mental disorders	++ [2 Pts]	++
Microcephaly	(+)	++
*Blood and bone marrow*		
Megaloblastic anaemia	+++ [5 Pts]	++
Pancytopenia/neutropenia	(+)	++
Recurrent severe infections	+++ [4 Pts]	(+)
*Kidneys*		
Haemolytic uraemic syndrome	++ [2 Pts]	++
Glomerulopathy	−	+
Tubulointerstitial nephropathy	−	+
*Cardiopulmonary*		
Cardiac malformation	++ [2 Pts]	+
Cardiomyopathy	(+)	++
Interstitial pneumonia	−	+
Pulmonary hypertension	−	+
*Vascular*		
Stroke	(+)	(+)
Venous thrombosis/embolism	(+)	+
*Malformations*		
Facial dysmorphism	++ [2 Pts]	+
Skeletal deformity	+++ [3 Pts]	(+)
*Gastrointestinal*		
Cheilitis/gastritis	(+)	−
Liver steatosis	−	+
*Skin*		
Dermatitis/rash/hyperpigmentation	−	+
*Other*		
Hydrops foetalis	−	+
Metabolic acidosis and/or hyperammonaemia	+++ [5 Pts]	+
Temperature instability/hypothermia	(+)	+

++++, very frequently (> 50% of cases);+++, frequently (25–50% of cases);++, infrequently (10–25% of cases);+, occasionally seen (< 10% of cases); (+) single case reports, probably disease-related conditions; -, absent/not reported; Pts, patients. The number of our *epi-cblC* patients who exhibited that sign/symptom is indicated in square brackets

^a^Clinical data for canonical-*cblC* patients (signs, symptoms and their frequencies) are derived from Huemer et al. [12]

## Discussion

Due to its prevalence, methylmalonic aciduria combined with homocystinuria type *cblC* is frequently identified by NBS[10]. Early detection of the disease allows prompt treatment and surveillance of affected newborns, which significantly reduces mortality and disease burden. For these reasons, *cblC* has been included in the Recommended Universal Screening Panel (RUSP) of the USA since 2006 [16]. In Italy, NBS for metabolic disorders has been recently regulated and made mandatory in all Italian regions (*Law n. 267/2016*). As a result, the number of *cblC* cases detected by NBS has increased.

When a combined methylmalonic aciduria with homocystinuria is suspected, genetic testing is needed to make differential diagnoses of the inherited *cbl* disorders which have been associated with at least 12 genes so far [17]. *MMACHC* is the most frequently mutated gene. However, a single heterozygous variant or no variants at all can be found in some patients with a *cblC* phenotype. Herein, we report the clinical, biochemical and molecular study of 11 *epi-cblC* probands who did not have a conclusive molecular diagnosis after routine *MMACHC* gene sequencing. In all patients the molecular diagnosis of *epi-cblC* disease was established by the identification of *MMACHC* epimutation and the related c.515-1G > T variant in the *PRDX1* gene. We classified ten patients with compound heterozygosity for the epimutation and a genetic variant of the *MMACHC* gene and one patient with a bi-allelic homozygous *MMACHC* epimutation due to the homozygous *PRDX1*:c.515-1G > T.

Until now, nine *epi-cblC* patients have been reported, all of whom with a mono-allelic *MMACHC* epimutation and a *MMACHC* genetic variant affecting the other allele [6, 18]. The *PRDX1*:c.515-1G > T variant was found in patients with Caucasian origin [6, 18] whereas the *PRDX1*:c.515-2A > T variant was only detected in one patient of Japanese-Korean ancestry [6]. To our knowledge, we are describing the first instance of *epi-cblC* due to a bi-allelic *MMACHC* epimutation.

Because of the limited number of *epi-cblC* patients described in the literature so far, epidemiological data about *epi-cblC* disease are lacking. Our retrospective study indicates that epi-*cblC* disease is more frequent than thought. It accounts for about 13% of NBS *cblC* patients diagnosed in Tuscany and Umbria (birth prevalence ~ 1:200.000) and for 11% of Italian *cblC* probands genetically characterized in our Unit. The *PRDX1* c.515-1G > T allele frequency currently reported in gnomAD (Genome Aggregation Database: https://gnomad.broadinstitute.org/) is 4.01e-5 (10/249150 European non-Finnish alleles, 5/10 Southern European). Our data show that this frequency is undoubtedly underestimated.

Genotype–phenotype correlations are possible in our patients. The *PRDX*1:c.515-1G > T variant leads to a loss of MMACHC protein expression through the secondary *MMACHC* epimutation triggered by its aberrant transcription [6]. Thus, an early- or late-onset phenotype in *epi-cblC* patients harbouring the heterozygous epimutation depends on the pathogenicity of the second *MMACHC* genetic variant. Consistently, our early onset *epi-cblC* cases had *MMACHC* truncating variants (nonsense or the c.271dupA) known to be associated with early onset *cblC* [11, 19]. In contrast, the adult *epi-cblC* patient in our study had the *MMACHC*:c.617G > A p.(Arg206Gln) variant. A previous study concluded that this variant was not associated with severe outcomes of *cblC* [14]. It suggested that it may play a role in variations of vitamin B12 and folates in the population without effecting the risk of developing diseases. Homozygosity for the *MMACHC*:c.617G > A substitution has not been reported and pathogenicity prediction tools (SIFT, Polyphen-2 and MutationTaster) classify it as damaging. The p.Arg206 is a conserved residue which belongs to an arginine-rich pocket close to the cobalamin binding site of the MMACHC protein [20]. These data suggest that the *MMACHC*:c.617G > A p.(Arg206Gln) is a disease-causing variant responsible for a mild structural alteration consistent with late-onset presentation. The adult patient has experienced many thrombotic manifestations, probably related to high levels of Hcy, which led to the misdiagnosis of homocystinuria and consequently non-optimal treatment. The *epi-cblC* case homozygous for the epimutation and the *PRDX1*:c.515-1G > T was detected by NBS. The newborn had an early onset form which was consistent with the silencing of *MMACHC* transcription evidenced by multiplex RT-PCR of fibroblast transcripts. Our findings indicate that the clinical signs and symptoms of *epi-cblC* are similar to that of canonical *cblC* disease. This is in line with previous reports on *epi-cblC* [6, 18]. A homozygous *PRDX1*:c.515-1G > T genotype seems to correlate with a more severe clinical phenotype than an homozygous *MMACHC*:c.271dupA genotype. As this observation is based on a unique *epi-cblC* homozygous patient, the identification and full characterization of additional *epi-cblC* homozygotes are needed to define the related phenotype. The early detection of a trough methionine level with a high peak of homocysteine in our *epi-cblC* homozygote could explain the more severe clinical phenotype in respect of the c.271dupA homozygotes used for comparison. In fact, it has been reported that a higher peak of homocysteine concomitant to a lower peak of methionine in the neonatal period correlates with poorer neurodevelopmental outcomes, because the damage could already arise in utero [13].

Despite clinical similarities between *epi-cblC* and canonical *cblC*, we cannot rule out that the functional consequences of the *PRDX1*:c.515-1G > T splicing variant on the PRDX1 protein have themselves an impact on the disease course in *epi-cblC* patients. PRDX1 encodes Peroxiredoxin 1, a versatile protein involved in cell defence against cellular oxidative stress with influences on cell growth, differentiation and apoptosis [21]. Its functions depend on subcellular localization (nucleus or cytosol), quaternary structure (homodimer, decamer or oligomer at high molecular weight) and environmental conditions. The main function of the PRDX1 protein is maintaining the intracellular reactive oxygen species (ROS) homeostasis by reducing hydrogen peroxide into water through a typical 2-cysteine catalytic mechanism [22]. Peroxide scavenging occurs at the expense of Cys52 oxidation through disulphide formation with the Cys173 residue from the other subunit [21]. The human Cys173 is located in the last exon of *PRDX1*, which is skipped due to the c.515-1G > T splicing variant [6]. Thus, an enzymatic PRDX1 defect is expected in our homozygous patient. In addition to its antioxidant function, PRDX1 is a transducer of redox signals [22], acts as a molecular chaperone of oligomers [23], enhances the natural killer cell cytotoxicity [24] and inhibits the function of oncoproteins such as c-Abl [25] and c-Myc [26]. The role of PRDX1 in suppressing tumours has been confirmed in *Prdx1*-knockout mouse models [27, 28]. *Prdx1*-knockout mice (*Prdx1*^*−/−*^) develop an age-related haemolytic anaemia, linked to higher levels of ROS in erythrocytes and various types of malignancies (lymphomas, sarcomas and carcinomas) associated with increased ROS-dependent DNA damage and functional c-Myc deregulation. Tumours arise both in *Prdx1*^*−/−*^ and *Prdx1*^±^ mice with an incidence ratio of 2:1 [27]. The molecular mechanisms of PRDX1-induced carcinogenesis are still not fully understood, although it has recently been reported that a *PRDX1* deletion leads to damage of telomeric DNA upon oxidative stress [29]. Although *PRDX1* gene variants have not been identified in human tumours [21], it is known that a tumorigenic human melanoma cell line, named MeWo-LC1, has a cellular phenotype identical to that of *cblC* patients’ cells. The growth of this cell line is methionine dependent, as a consequence of the epigenetic inactivation of *MMACHC* gene through *PRDX1* aberrant transcription [30]. For these reasons, we recommend periodically monitoring subjects bearing the c.515-1G > T variant to prevent the potential risk of cancer.

According to the criteria that define the types of epimutations [31, 32], *epi-cblC* results from a secondary constitutional epimutation, which produces the phenotype of the disease. The epimutation is the consequence of a nearby *PRDX1* sequence alteration that produces an aberrant antisense transcription encompassing the *MMACHC* promoter [6]. An epigenetic silencing caused by aberrant transcription (i.e. transcriptional interference) has been previously described for Lynch syndrome (*MSH2* gene) [33] and α-thalassaemia (*HBA2* gene) [34]. Transcriptional interference arises at tandem and convergent promoters and involves different mechanisms, e.g. promoter occlusion, roadblocking and RNA polymerase collision [35]. The result of these events is DNA methylation, but the involved processes and factors are not entirely clear [36]. In these three diseases, the abolishment of transcription termination and concomitant transcriptional read-through lead to methylation of the CpG island and silencing of the downstream gene, irrespective of the orientation of the latter. In *epi-cblC* disease, the loss of poly(A) signal is due to a *PRDX1* splice site variant which causes skipping of its last exon and aberrant antisense transcription [6]. In both α-thalassaemia and Lynch syndrome, the causes of transcriptional elongation are genomic deletions. Specifically, in α-thalassaemia the genomic deletion in the 3′-end of forward *LUC7L* gene produces an elongated transcript that overlaps the *HBA2* reverse gene [34]. In Lynch syndrome, terminal deletions of forward *EPCAM* gene result in transcriptional read-through across the *MSH2* promoter in sense direction [33]. A difference between epimutations of *MMACHC* and *HBA2* concerns their maintaining or not in germ cells. In sperm, the *MMACHC* secondary epimutation is maintained [6], while the epigenetic silencing of *HBA2* is erased [34, 37].

The secondary epimutation of *epi-cblC* patients is located in a reverse *CCDC163P*-forward *MMACHC*-reverse *PRDX1* trio of genes (R1-F2-R3) [6]. Gueant et al. [6] reported that similar configuration of gene trios exists in other regions of the human genome and provided a list of such trios. Thus, on a broader perspective, this epigenetic mechanism could be associated with other IEMs, as for example: Sandhoff disease (OMIM #268800), *ACAT2* deficiency (OMIM #203750), Mucolipidosis III gamma (OMIM #252605), cystinosis (OMIM #219800, 219750 and 219900) and galactosialidosis (OMIM #256540). This finding should be kept in mind when routine molecular analysis results inconclusive in patients with a clear-cut clinical/biochemical phenotype.

## Conclusions

In summary, we found that *epi-cblC* disease caused by the *MMACHC* epimutation secondary to the *PRDX1*:c.515-1G > T variant is a relatively frequent inborn error of cobalamin metabolism and report the first instance of *epi-cblC* due to a bi-allelic *MMACHC* epimutation. *MMACHC* epimutation/*PRDX1* mutation analyses should be part of routine genetic testing for all patients presenting with a metabolic phenotype that combines methylmalonic aciduria and homocystinuria. Future research could elucidate the epidemiology, pathophysiology and prognosis of *epi-cblC* patients.

## Methods

### Patients

Eleven patients referred from several Italian regions to our diagnostic laboratory between 2008 and 2018 were included in this study. All patients received a diagnosis of methylmalonic aciduria and homocystinuria by NBS or after a clinical/biochemical assessment. At the time of diagnosis, seven patients were neonates (up to 1 month of age), three were young infants (2–6 months old) and one was an adult (63 years old). Two patients (Pts 1 and 2) had previously been described due to their ocular manifestations [38]. Mutation and epimutation analysis of the *MMACHC* gene for the adult patient (Pt 10) was previously reported [6]; herein, we describe his medical history and genotype–phenotype correlation. The study was performed in line with the principles of the 1964 Helsinki Declaration and approval was granted by the Ethics Committee of the Tuscany Region (No. CS_01/2021). Informed consent was obtained from all individual participants included in the study.

### Biochemical analysis

For the cases detected by NBS, diagnosis was initially made by identifying increased levels of C3 and reduced Met. As for clinically diagnosed patients, confirmatory laboratory testing was performed on all positive NBS cases. Tests we carried out included plasma and/or urinary amino acid analysis, determination of plasma homocysteine, plasma and/or urinary organic acid analysis and/or plasma acylcarnitine analysis. In four patients (Pts 1, 2, 10, and 11) specialized biochemical assays for the cobalamin metabolic pathway were performed on cultured fibroblasts in the laboratory of Prof. Matthias Baumgartner (University Children's Hospital, Zurich, Switzerland).

### Sequencing analysis

Genomic DNA of the patients and their parents was extracted from peripheral blood using a QIAsymphony instrument (Qiagen, Hilden, Germany). We performed Sanger sequencing of the *MMACHC* gene in all patients, as previously reported [38]. In patients 9–11, we also performed targeted NGS of 40 genes linked to methylmalonic aciduria, hyperhomocysteinemia and disorders of cobalamin and folate metabolism using a custom-designed panel (Illumina, San Diego, CA). We prepared libraries using the Nextera rapid capture enrichment kit (Illumina) according to the manual instructions. The libraries were sequenced by a paired-end 2 × 150 bp protocol on a MiSeq System (Illumina, San Diego, CA) to obtain an average coverage of above 100x, with > 95% of target bases covered at least 15x. For data analysis, we used the BWA, Picard and GATK tools. Variant annotation was performed by the ANNOVAR tool. To identify possible CNVs, the sequencing data were analysed by the CoNVaDING tool [39]. Screening of the known c.515-1G > T and c.515-2A > T variants in the *PRDX1* gene was performed on genomic DNA by Sanger sequencing of the specific intron–exon boundary (intron 6-exon 7). This boundary was amplified by PCR using the primer pair PRDX1-7fw and PRDX1-7rev (Additional file 1: Table S1), appropriately designed to avoid an artefact due to the presence of a poly-T after the termination codon in sequencing analysis. PCR products were sequenced with the same primers by using the BigDye Terminator v1.1 Cycle Sequencing Kit and an ABI PRISM 3130 GeneticAnalyser (Applied Biosystems, Foster City, CA, USA). The reference sequences used for nomenclature of the *MMACHC* and *PRDX1* variants were NM_015506.3 and NM_002574.3, respectively.

### *MMACHC* expression analysis in the homozygous* PRDX1* patient

To demonstrate a transcriptional silencing of the *MMACHC* gene caused by the *PRDX1*:c.515-1G > T variant in the homozygous patient (Pt 11), we performed a non-quantitative reverse transcription-polymerase chain reaction (RT-PCR) assay. Total RNA was obtained from cultured fibroblasts of the patient and two normal controls with RNeasy Protect Mini Kit (Qiagen, Hilden, Germany). cDNA was produced using the random hexamers and the TaqMan Reverse Transcription kit (Applied Biosystems). A multiplex PCR assay was performed to simultaneously amplify a fragment of 512 bp for the *MMACHC* gene and a fragment of 174 bp for the *ACTB* gene, used as a housekeeping gene. Primers for multiplex RT-PCR and PCR conditions are listed in Additional file 1: Table S1. PCR products were checked by agarose gel electrophoresis.

### DNA methylome analysis

To confirm that the *PRDX1*:c.515-1G > T variant identified in our patients produces an epimutation in the *MMACHC* promoter, we performed an epigenome-wide DNA methylome analysis, as previously described [6], in the *epi-cblC* patients carrying the heterozygous *PRDX1*:c.515-1G > T variant. The same analysis was performed in the proband (Pt 11) carrying the *PRDX1*:c.515-1G > T at a homozygous level and his parents. We carried out a bisulphite conversion of 600 ng of DNA extracted from whole blood using the EZ DNA Methylation kit (Zymo Research, Proteigene, Saint-Marcel, France). Genome-wide profiling of DNA methylome was determined using the Infinium MethylationEPIC BeadChip array (Illumina, Paris, France), according to the manufacturer’s instructions. The Infinium MethylationEPIC BeadChip provides a coverage of 850,000 CpG probes in enhancer regions, gene bodies, promoters, and CpG islands. The arrays were scanned on an Illumina iScan® system, and raw methylation data were extracted using the Illumina GenomeStudio Methylation Module. For each CpG probe, the methylation level was described as a β value, ranging between 0 (fully unmethylated CpG probe) and 1 (fully methylated CpG probe). Background correction and normalization was implemented using the SWAN method (R Package Minfi) [40]. We visually inspected the whole-genome distribution of the CpG probes according to their β values. In the epigenome-wide association study, we compared the whole blood DNA methylome profile of subjects with *epi-cblC* with 350 controls from the MARTHA cohort [41]. For each CpG probe, we compared the mean β values between *epi-cblC* subjects and controls using a *t*-test with Bonferroni and false discovery rate corrections to account for multiple testing. Due to the low sample size, and considering the exploratory approach of our analysis, we used the smoothed *P*-value transformation by converting nominal *P*-values obtained from the *t*-test to smoothed *P*-values using a window radius of 3, as previously reported [6]. All statistical analyses were performed using the SNP & Variation Suite (v8.8.1; Golden Helix, Inc., Bozeman, MT, USA).

### Estimation of *epi-cblC* prevalence and *PRDX1* c.515-1G > T allele frequency

The NBS-case cohort of the Tuscany and Umbria regions was used to estimate the birth prevalence of *epi-cblC* disease. Instead, to estimate the frequency of the *PRDX1*:c.515-1G > T mutant allele among *cblC* patients, we used the total *cblC*-case cohort which includes all patients with a confirmed molecular diagnosis analysed in our laboratory since 2006. We divided the *cblC* patients into three groups: *MMACHC-*cases or “canonical-*cblC*” with bi-allelic genetic *MMACHC* variants, *MMACHC/PRDX1-*cases with compound heterozygosity for a *MMACHC* genetic variant and an epigenetic variant, and *PRDX1*-cases or “homozygous *epi-cblC*” with the *PRDX1*:c.515-1G > T variant at a homozygous level leading to bi-allelic *MMACHC* epimutation.

### Comparison of clinical phenotype between *epi-cblC* and canonical *cblC* patients

To evaluate if the clinical phenotype of *epi-cblC* is similar to that of canonical *cblC*, we first compared signs and symptoms between *epi-cblC* and *cblC* patients according to a detailed review on *cblC* presentation [12]. Then, we compared NBS data and clinical manifestations of our *epi-cblC* homozygous patient with *MMACHC:*c.271dupA homozygous patients from the Tuscany-Umbria NBS cohort, who we deemed representative of early onset canonical *cblC.*

## Supplementary Information

**Additional file 1.** **Table S1.** Primers used for *PRDX1* mutational analysis and multiplex RT-PCR of the *MMACHC* gene. **Table S2.** Clinical findings of patients with *epi-cblC* disease. **Table S3.** Metabolic findings at diagnosis of patients with *epi-cblC* disease. **Table S4.** *PRDX1*:c.515-1G>T homozygosity vs *MMACHC*:c.271dupA homozygosity: metabolic and clinical findings from the Tuscany/Umbria NBS program. **Figure S1.** Density distribution plot of the methylome profiles assayed by the Infinium MethylationEPIC BeadChip array in the analysed subjects. All the DNA methylome profiles had a density distribution that followed a beta distribution and were included in the analysis. **Figure S2.** Estimation of *epi-cblC* prevalence and *PRDX1* mutant allele frequency. **a** Distribution of *cblC* diagnoses in the Tuscany-Umbria NBS-case cohort and birth prevalence of *cblC* and *epi-cblC* diseases. Three different subgroups of patients have been distinguished on the basis of gene/genes mutated. *MMACHC* and *MMACHC/PRDX1* cases are coloured in blue and orange, respectively. The *PRDX1*-case with the bi-allelic *PRDX1*:c.515-1G>T variant, is in red. Values in the pie chart indicate the number and percentage of cases belonging to each subgroup. **b** Distribution of *cblC* diagnoses in the total *cblC*-case cohort referred for genetic test to our Unit since 2006. **c** Allele frequencies of the most common *cblC* disease-causing variants identified in the total *cblC*-case cohort. The *MMACHC* genetic variants are coloured in blue, whereas the *PRDX1* epimutation is in red.

## Data Availability

All data generated or analysed during this study are included in this published article and its supplementary information files, or they are available from the corresponding author on reasonable request.

## Abbreviations

AdoCbl: Adenosylcobalamin
C2: Acetylcarnitine
C3: Propionylcarnitine
cbl: Cobalamin
CNVs: Copy number variations
Hcy: Homocysteine
MeCbl: Methylcobalamin
Met: Methionine
MMA: Methylmalonic acid
NBS: Newborn screening
NGS: Next-generation sequencing
ROS: Reactive oxygen species
RUSP: Recommended universal screening panel
