# Supplementary material for: PRDX1 gene-related epi-cblC disease is a common type of inborn error of cobalamin metabolism with mono- or bi-allelic MMACHC epimutations
Source: Clin Epigenetics. 2021 Jul 2;13:137. doi: 10.1186/s13148-021-01117-2 (PMC8254308; doi:10.1186/s13148-021-01117-2)
Supplement: Supplementary file 1 — Additional file 1. Table S1. Primers used for PRDX1 mutational analysis and multiplex RT-PCR of the MMACHC gene. Table S2. Clinical findings of patients with epi-cblC disease. Table S3. Metabolic findings at diagnosis of patients with epi-cblC disease. Table S4. PRDX1:c.515-1G>T homozygosity vs MMACHC:c.271dupA homozygosity: metabolic and clinical findings from the Tuscany/Umbria NBS program. Figure S1. Density distribution plot of the methylome profiles assayed by the Infinium MethylationEPIC BeadChip array in the analysed subjects. All the DNA methylome profiles had a density distribution that followed a beta distribution and were included in the analysis. Figure S2. Estimation of epi-cblC prevalence and PRDX1 mutant allele frequency. a Distribution of cblC diagnoses in the Tuscany-Umbria NBS-case cohort and birth prevalence of cblC and epi-cblC diseases. Three different subgroups of patients have been distinguished on the basis of gene/genes mutated. MMACHC and MMACHC/PRDX1 cases are coloured in blue and orange, respectively. The PRDX1-case with the bi-allelic PRDX1:c.515-1G>T variant, is in red. Values in the pie chart indicate the number and percentage of cases belonging to each subgroup. b Distribution of cblC diagnoses in the total cblC-case cohort referred for genetic test to our Unit since 2006. c Allele frequencies of the most common cblC disease-causing variants identified in the total cblC-case cohort. The MMACHC genetic variants are coloured in blue, whereas the PRDX1 epimutation is in red. [file 13148_2021_1117_MOESM1_ESM.doc]

**Supplemental Materials**

***PRDX1* gene-related *epi-cblC* disease is a common type of inborn error of cobalamin metabolism with mono or bi-allelic *MMACHC* epimutations**

**Table of Contents**

**Supplemental Table S1**

**Supplemental Table S2**

**Supplemental Table S3**

**Supplemental Table S4**

**Supplemental Figure S1**

**Supplemental Figure S2**

**Table S1. Primers used for *PRDX1* mutational analysis and multiplex RT-PCR of the *MMACHC* gene**

| **Application (sample)** | **Primer pair** | **Direction** | **Sequences (5’-3’)** | **Location** | **Reference sequence** | **Fragment length (bp)** | **PCR conditions** |
| --- | --- | --- | --- | --- | --- | --- | --- |
| **Detection of the *PRDX1* c.515-1G>T and c.515-2A>T variants (gDNA)** | PRDX1-7fw  PRDX1-7rev | Forward  Reverse | TTAAGGATTAGAGAGTGTTGGCA  TTTTGTTCTCATGGCTGCCC | Intron 6  3’UTR | NM_002574.3 | 431 | 95°C 5 min; 30 cycles consisting of 95°C 30 s, 58°C 30 s and 72°C 45 s; final extension at 72°C 7 min |
| **Multiplex RT-PCR* (cDNA)** | MMACHC-Afw  MMACHC-Brev  ACTB-fw  ACTB-rev | Forward  Reverse  Forward  Reverse | CAGCAAGCTCAGCGTGTAAC  CTGATATGCGCTGGTTCCC  AGCCTCGCCTTTGCCGA  CTGGTGCCTGGGGCG | Exon 1  Exon 3-Exon 4  Exon 1  Exon 2 | NM_015506.2  NM_001101.4 | 512  174 | 95°C 5 min; 30 cycles consisting of 95°C 30 s, 58°C 30 s and 72°C 45 s; final extension at 72°C 7 min |

gDNA: genomic DNA; cDNA: complementary DNA. **ACTB* gene, encoding the actin beta protein, was used as the housekeeping gene in multiplex RT-PCR assay

**Table S2. Clinical findings of patients with *epi-cblC* disease**

| **Pt (sex)** | **Diagnosis (age)** | **Perinatal findings** | **Signs and symptoms at onset/diagnosis** | **Later signs and symptoms** | **Treatments** | **Clinical outcome** |
| --- | --- | --- | --- | --- | --- | --- |
| **1 (M)** | C/B  (10 d) | Caesarean delivery at 36 w due to placental abruption, BW 2020 g, BL 44 cm, OFC 29 cm, Apgar score 5-8 | Drowsiness, hypotonia, hypoglicemy and sucking difficulties (2 d); hyperalaninemia detected by NBS (6 d); inefficient thermoregulation (8-20 d) | Recurrent vomiting (3-4 m); maculopathy, nystagmus and strabismus (7 m); three episodes of acute gastroenteritis (1 y, 6 y and 7 y); psychomotor delay, speech delay, hyperactivity and severe low vision (2-3 y); bilateral ventricular dysmorphism and thinning of the corpus callosum with normal spectroscopy (MRI, 7 y) | Enteral feeding (2-20 d); incubator care (8-20 d); OH-cbl, L-carnitine (30 d); betaine and folates (40 d); ranitidine (3 m); lansoprazole and domperidone (6 m); antibiotics (6-7 y) | Alive  (10 y) |
| **2 (M)** | NBS  (6 d) | Born at 39 + 6 w, BW 3066 g, BL 51 cm, OFC 34 cm, Apgar score 9-10 | Jaundice, dysmorphic features (small chin and long fingers) (1 d); poor feeding, jerky spontaneous movements, anaemia and metabolic acidosis (6 d) | Hypotonia (1 m and 20 d); nystagmus (7 m); maculopathy (9 m); recurrent infections and febrile seizures (1-2 y); psychomotor delay (1 y); severe low vision (3 y); speech delay (4 y); mild ventricular enlargement with normal spectroscopy (MRI, 7 y) | Enteral feeding (6-10 d); OH-cbl, L-carnitine, betaine and folates (6 d); antibiotics (1-2 y) | Alive  (8 y) |
| **3 (M)** | C/B  (16 d) | IUGR, born at 34 + 4 w due to and acute foetal distress, BW 1400 g, Apgar score 7-8 | Anaemia and respiratory distress (2 d); metabolic acidosis, leukopenia, severe neutropenia and thrombocytopenia (8 d); small-sized ventricular septal defect and medium-sized atrial septal defect (15 d); germinal matrix hyperechogenicity (MRI, 16 d) | Urinary tract infection (22 d); speech delay and mild cognitive deficit, nystagmus and retinopathy (4 y) | NCPAP (2 d, 7-16 d); parenteral nutrition (9-15 d); enteral feeding (16-38 d); repeated platelet transfusions (9-39 d); red blood cell transfusions (9-81d); filgrastim therapy (16-58 d); liposomal amphotericin B and 5-fluorocytosine (from 22 d); OH-cbl, L-carnitine, betaine and folates (38 d) | Alive  (11 y) |
| **4 (M)** | C/B  (2 m) | na | Recurrent vomiting, failure to thrive, hypotonia, lethargy, tachypnea and metabolic acidosis (2 m) | na | na | Died  (2 m) |
| **5 (M)** | C/B  (2 m) | Born at term, BW 3080 g, BL 48 cm, OFC 32.5 cm and Apgar score 9 | Sucking difficulties, recurrent vomiting, microcephaly, dolichocephaly and micrognathia (4 d); severe weight loss (21 d); hospitalization because of severe failure to thrive and vomiting with subsequent drowsiness, hypotonia, sepsis, respiratory distress and acute neurological crisis (42-104 d); frontotemporal atrophy with hygromas (MRI, 2m) | Nystagmus (4 m); moderate mental delay (3 y); macular hypoplasia (6 y); exotropia/exophoria (7 y) | Enteral feeding, red blood cell transfusions, OH-cbl L-carnitine, betaine and folates (2 m); antibiotics, antifungal drugs and NCPAP during the episode of sepis (42-104 d) | Alive  (10 y) |
| **6 (M)** | C/B  (6 m) | Born at 36 + 3 w, BW 2020 g, BL 45 cm, OFC 31 cm and Apgar score 9 | Respiratory distress, jaundice, hypotonia and sucking difficulties (1 d); gastroenteritis (1 m); motor delay (4 m); mild failure to thrive, anaemia, poor visual fixation, delay in visual maturation and ERG abnormalities (6 m) | Thinning of the corpus callosum and delayed myelination (MRI, 9 m); seizures (10 m); maculopathy (1 y); exotropia (14 m); multifocal EEG abnormalities (4 y); seizures (5 y); intracerebral cyst in globus pallidus (MRI, 6 y) | Phototherapy for jaundice (1 d); enteral feeding (2-12 d); OH-cbl, L-carnitine, betaine and folates (6 m); iron (6-9 m); nitrazepam (10-11 m); iron (3-4 y); valproate (5 y) | Alive  (6 y) |
| **7 (F)** | NBS  (1 m) | Born at 36 w (triplet pregnancy) and BW 1920 g | Acute urinary tract infection, poor feeding and failure to thrive (1 m) | Psychomotor delay (inability to grasp objects as first sign) and nystagmus (3 y); dysarthria (4 y); improvement in nystagmus and dysarthria (5 y); maculopathy with severe visual loss and attention-deficit/hyperactivity disorder (6 y) | OH-cbl, L-carnitine, betaine and folates (1 m) | Alive  (7 y) |
| **8 (M)** | C/B  (1 m) | Born at term, BW 3150 g, BL 51cm, OFC 34 cm and Apgar score 10 | Recurrent vomiting, failure to thrive, hypotonia, metabolic acidosis and HUS (1 m) | Language delay, cognitive delay and learning disabilities (3 y); strabismus, nystagmus, maculopathy and ERG abnormalities (first recorded 11 y); acute neurological crisis (drooling, eye-rolling back and generalized tonic-clonic movements with normal EEG) (14 y); kyphoscoliosis, pes planus, hypercholesterolemia and [hypertriglyceridemia](https://www.google.com/url?sa=t&rct=j&q=&esrc=s&source=web&cd=1&cad=rja&uact=8&ved=2ahUKEwipr57AjKvnAhUSzaQKHaEcDScQFjAAegQIAhAB&url=https%3A%2F%2Femedicine.medscape.com%2Farticle%2F126568-overview&usg=AOvVaw0xRQk3n_vZvA_03mAfyCgh) (17 y) | CVVHD, OH-cbl, L-carnitine, betaine and folates (1 m); valproic acid (14 y, for 8 m) then substituted for carbamazepine; hypolipidemic diet and cholecalciferol (17 y) | Alive  (19 y) |
| **9 (F)** | NBS  (4 d) | IUGR, born at 38 + 6 w, BW 2045 g, BL 44 cm, OFC 29.5 cm, Apgar score 5-9 | Bradycardia, hypotonia and cyanosis (1 d); HUS (3 d); failure to thrive and left ventricular hypertrabeculation with normal systolic function (4 d) | Maculopathy (3 m); strabismus (8 m); VEP and ERG abnormalities (9 m); mild developmental delay (13 m) | NCPAP (1 d); OH-cbl and L-carnitine (4 d); betaine and folates (5 d) | Alive  (2 y) |
| **10 (M)** | C/B  (63 y) | Labour dystocia due to podalic presentation | Limb and abdomen pains (3-10 y); acute nephritis and anaemia (29 y); membranoproliferative glomerulonephritis and xerophthalmia secondary to SLE (30 y); hypertension (38 y); dysphonia (44 y); paroxysmal atrial fibrillation (45 y); deep venous thrombosis (49 y); sensorimotor polyneuropathy and antiphospholipid syndrome (50 y); pulmonary thromboembolism and homocystinuria (53 y); several transient ischemic attacks (56-60 y); distal tremors (60 y); subarachnoid haemorrhage (61 y); thrombosis of left renal artery (62 y); APCA positivity (63 y) | Chronic gastritis and dolichocolon with colic melanosis (64 y); asthenia and severe back and limb pains (65 y); xerophthalmia (68 y); second episode of paroxysmal atrial fibrillation (70 y); deterioration of renal function, diffuse hypotonia, worsening of polyneuropathy with motor coordination deficit and white matter lesions at MRI compatible with gliosis and vasculitis (71 y) | Antihypertensive agents (38 y); antiarrhythmics (45 y); steroids (50 y); anticoagulants, pyridoxine, folates and OH-cbl (53 y); interruption of treatments due to unresponsiveness of homocystinuria (55-59 y); antiplatelet therapy, pyridoxine, folates and OH-cbl (60 y); neurosurgical treatment for subarachnoid haemorrhage (61 y); betaine and OH-cbl (63 y); pregabalin (63-65 y); opioids (65 y); higher-dose OH-cbl therapy (71 y); discontinuation of betaine (72 y); reduced dosage of folates (73 y) | Alive  (75 y) |
| **11 (M)** | NBS  (4 d) | Born at 39 + 3 w, BW 3300 g, OFC 34 cm, Apgar score 8-9 and hypospadias | Apnoea, cyanosis and severe hypotonia (1 d); poor feeding, vomiting, small-sized ventricular septal defect and ECG abnormalities (4 d) | Anaemia and thrombocytopenia (10 d); respiratory distress due to bronchopneumonia (13 d); strabismus and nystagmus (4 m); severe gastroenteritis, fever, seizures and metabolic acidosis (6 m); maculopathy (7 m); pyelonephritis, fever, seizures and psychomotor delay (1 y); cerebellar white matter hyperintensities with normal spectroscopy (1 y and 3 m); speech delay and visual deterioration (3 y); autism spectrum disorder (4 y) | Enteral feeding (4-16 d); OH-cbl, L-carnitine, betaine and folates (4 d); [benzodiazepine](https://en.wikipedia.org/wiki/Benzodiazepine) (6 m); [corrective lenses](https://en.wikipedia.org/wiki/Corrective_lens) and [eye patch](https://en.wikipedia.org/wiki/Eye_patch) (7 m) | Alive  (7 y) |

APCA: anti-parietal cell antibodies; BL: birth length; BW: birth weight; C/B: diagnosis of methylmalonic aciduria and homocystinuria performed after a clinical/biochemical assessment; CVVHD: continuous veno-venous haemodialysis; d: days; ECG: electrocardiogram; EEG: electroencephalography; ERG: electroretinography; F: female; HUS: haemolytic uremic syndrome; IUGR: intrauterine growth restriction; m: months; M: male; MRI: magnetic resonance imaging; na: not available; NBS: diagnosis of methylmalonic aciduria and homocystinuria performed by expanded newborn screening; NCPAP: nasal continuous positive airway pressure; OH-cbl: hydroxycobalamin; OFC: occipitofrontal circumference at birth; SLE: systemic lupus erythematosus; VEP: visual evoked potential; w: weeks; y: years.

**Table S3. Metabolic findings at diagnosis of patients with *epi-cblC* disease**

| **Patient** | **Diagnosis** | **C3 on DBS (μmol/l)** | **Met on DBS (μmol/l)** | **MMA on DBS (μmol/l)** | **Plasma C3 (μmol/l)** | **Plasma MMA (μmol/l)** | **Plasma Hcy (μmol/l)** | **Plasma Met (μmol/l)** | **Urine MMA (mmol/mol creat)** |
| --- | --- | --- | --- | --- | --- | --- | --- | --- | --- |
| **1^a^** | C/B | 3 (n.v. <3.3) | 27.4 (n.v. 6-36) | increased at 10 d of life | 12.6 (n.v. <1) | na | 184 (n.v. <15) | na | 1468 (n.v. <2) |
| **2** | NBS | 7 (n.v. <3.3) | 5.6 (n.v. 6-36) | 44 (n.v. <2) | 11 (n.v. <1) | na | 183 (n.v. <15) | 9 (n.v. 15-36) | 388 (n.v. <2) |
| **3*^b^*** | C/B | increased | na | na | increased | increased | 93.2 (n.v. 5-15) | 0 (n.v 6-36) | 5652 (n.v <2) |
| **4** | C/B | no NBS | no NBS | no NBS | na | na | increased | decreased | increased |
| **5** | C/B | no NBS | no NBS | no NBS | na | na | 109 (n.v <15) | 10 (n.v. 15-21) | 2812 (n.v. < 2) |
| **6** | C/B | no NBS | no NBS | no NBS | na | na | 174 (n.v <15) | 10 (n.v. 15-21) | 2570 (n.v. < 2) |
| **7** | NBS | 11.6 (n.v <4.8) | 4.9 (n.v. 6.7-28.6) | na | na | na | 67 (n.v. <13) | 18 (n.v. 22-32) | increased |
| **8** | C/B | no NBS | no NBS | no NBS | na | na | increased | decreased | increased |
| **9** | NBS | 12.3 (n.v. <4.5) | 5.9 (n.v. <8.3) | 214.5 (n.v. <1.1) | 16.5 (n.v. <1) | 521 (n.v. <4) | 153 (n.v. 4-15.4) | 7 (n.v. 15-21) | >6000 (n.v. <2) |
| **10** | C/B | no NBS | no NBS | no NBS | na | increased | 357 (n.v. <15) | 15 (n.v. 6-40) | 2706 (n.v. <2) |
| **11** | NBS | 10.3 (n.v. <3.3) | 5.8 (n.v. 6-36) | 155 (n.v. <2) | 11.5 (n.v. <1) | 1490 (n.v. <1) | 205 (n.v. <15) | 2.6 (n.v. 15-39) | 1545 (n.v. <2) |

C/B: diagnosis performed after a clinical/biochemical assessment; C3: propionylcarnitine; creat: creatinine; d: days; DBS: dried blood spot; Hcy: homocysteine; Met: methionine; MMA: methylmalonic acid; na: not available; NBS: diagnosis performed by expanded newborn screening; n.v.: normal values; y: years.

^a^This patient showed normal values of C3, methionine and marker ratios at NBS, but an unusually high level of alanine was detected (862 μmol/l, n.v. <300). As alanine is not a biomarker for NBS, he was not initially recalled. Because of early symptoms, we carried out additional metabolic tests which showed normal blood lactate and increased levels of both MMA and homocystine in urine.

^b^This patient showed symptoms before NBS result was available

**Table S4. *PRDX1*:c.515-1G>T homozygosity *vs* *MMACHC*:c.271dupA homozygosity: metabolic and clinical findings from the Tuscany/Umbria NBS program**

|  |  |  | **NBS values** | | | **Confirmatory tests** | | | **Clinical findings** | | | | | | | | | | |
| --- | --- | --- | --- | --- | --- | --- | --- | --- | --- | --- | --- | --- | --- | --- | --- | --- | --- | --- | --- |
| **Pt** | **Birth year** | **Homozygous variant** | **C3**  μmol/l  n.v. <3.3 | **Met**  μmol/l  n.v. 6-36 | **MMA**  μmol/l  n.v. <2 | **Hcy^P^**  μmol/l  n.v. <15 | **Met^P^**  μmol/l  n.v. 15-39 | **MMA^U^**  mmol/mol creat  n.v. <2 | **FTT** | **NH** | **AMC** | **HEM** | **HUS** | **M** | **S** | **DD** | **BMD** | **CVD** | **BI** |
| Pt 11 | 2013 | *PRDX1*:c.515-1G>T | 10.3 | 5.8 | 155 | 205 | 2.6 | 1545 | + | + | + | + | - | + | + | + | + | + | + |
| (1)^a^ | 2017 | *MMACHC*:c.271dupA | 11.6 | 14.3 | 344 | 138 | 9.9 | 2685 | - | - | - | - | - | + | - | - | - | + | - |
| (2) | 2016 | *MMACHC*:c.271dupA | 6.9 | 5.4 | 57 | 207 | 8.7 | 1428 | - | - | - | - | - | + | - | - | - | + | - |
| (3) | 2014 | *MMACHC*:c.271dupA | 7.1 | 4.9 | 29.6 | 264 | 6 | 111^c^ | + | + | - | - | - | + | - | + | + | + | + |
| (4) | 2012 | *MMACHC*:c.271dupA | 7.9 | 5.1 | 29.5 | 120 | 6.2 | 576 | - | - | - | - | - | - | - | + | - | - | + |
| (5) | 2011 | *MMACHC*:c.271dupA | 6.7 | 6.5 | Yes^b^ | 127 | 6.9 | 1696 | + | + | - | - | - | + | - | - | - | - | - |
| (6) | 2010 | *MMACHC*:c.271dupA | 5.3 | 5 | Yes^b^ | 236 | 6.1 | 1251 | - | + | + | - | - | + | - | + | + | - | + |
| (7) | 2020 | *MMACHC*:c.271dupA | 6.9 | 8.2 | 274 | 95.4 | 5.9 | 770 | - | + | - | - | - | - | - | - | - | + | - |

**^P^**: plasma; **^U^**: urine; FTT: failure to thrive failure; NH: neonatal hypotonia; AMC: acute metabolic crises; HEM: haematological abnormalities; HUS: haemolytic uremic syndrome; M: maculopathy; S: seizures; DD: developmental disorder/cognitive disorder; BMD: behavioural/mental disorder; CVD: cardiovascular disorder; BI: brain imaging abnormalities; +: present; -: absent.

^a^This patient had a low birth weight (1660 g) and needed total parenteral nutrition.

^b^MMA determination was performed by a qualitative test.

^c^First measurement after therapy was started.

*
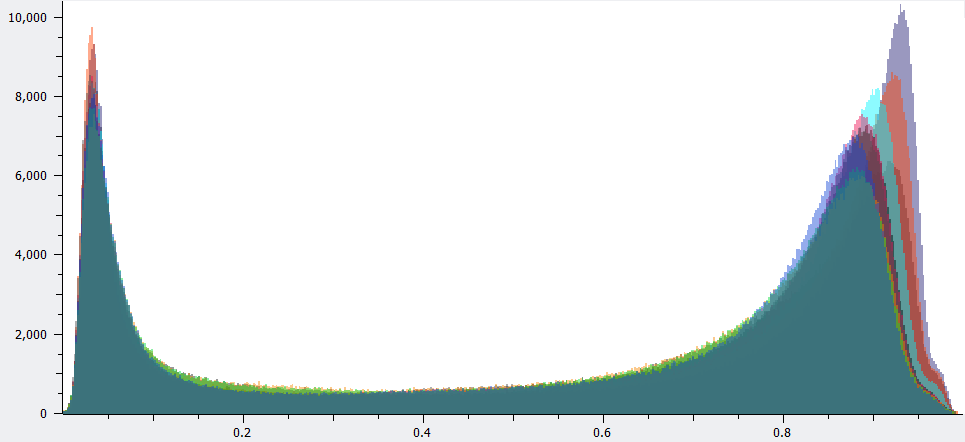
*

**Figure S1. Density distribution plot of the methylome profiles assayed by the Infinium MethylationEPIC BeadChip array in the analysed subjects**

All the DNA methylome profiles had a density distribution that followed a beta distribution and were included in the analysis


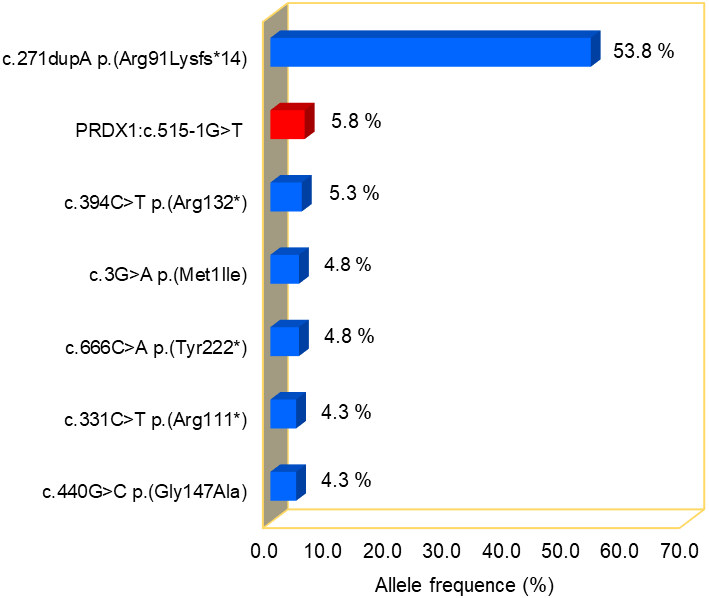


***PRDX1*:c.515-1G>T**

**a**

**c**

**Total cblC-case cohort (N= 104 probands)**

**b**


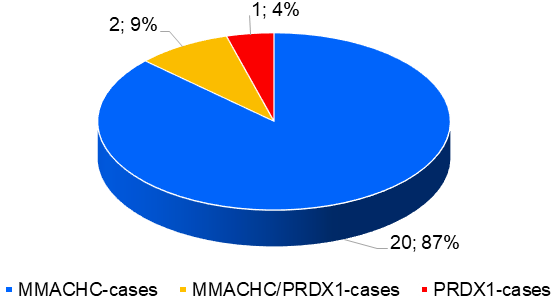


**Tuscany-Umbria NBS-case cohort (N= 600,387 newborns)**


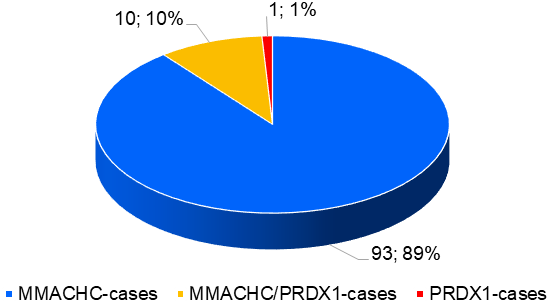


**Total cblC-case cohort (N= 104 probands)**

Birth prevalence

cblC disease 1:26,000

epi-cblC disease 1:200,000

23 *cblC* cases

3 *epi-cblC* cases

**Figure S2. Estimation of *epi-cblC* prevalence and *PRDX1* mutant allele frequency**

**a** Distribution of *cblC* diagnoses in the Tuscany-Umbria NBS-case cohort and birth prevalence of *cblC* and *epi-cblC* diseases. Three different subgroups of patients have been distinguished on the basis of gene/genes mutated. *MMACHC* and *MMACHC*/*PRDX1* cases are coloured in blue and orange, respectively. The *PRDX1*-case with the bi-allelic *PRDX1*:c.515-1G>T variant, is in red. Values in the [pie chart](https://en.wikipedia.org/wiki/Pie_chart) indicate the number and percentage of cases belonging to each subgroup. **b** Distribution of *cblC* diagnoses in the total *cblC*-case cohort referred for genetic test to our Unit since 2006. **c** Allele frequencies of the most common *cblC* disease-causing variants identified in the total *cblC*-case cohort. The *MMACHC* genetic variants are coloured in blue, whereas the *PRDX1* epimutation is in red
